# The therapeutic role of γδT cells in TNBC

**DOI:** 10.3389/fimmu.2024.1420107

**Published:** 2024-06-12

**Authors:** Wenjing Li, Xian Zhao, Chuanxin Ren, Shang Gao, Qinyu Han, Min Lu, Xiangqi Li

**Affiliations:** ^1^ Department of Breast Center, The Second Affiliated Hospital of Shandong First Medical University, Tai’an, Shandong, China; ^2^ Department of The First Clinical Medical School, Shandong University of Traditional Chinese Medicine, Jinan, Shandong, China

**Keywords:** γδT cell, gamma delta T cell, TNBC, immunotherapy, breast cancer

## Abstract

Triple-negative breast cancer (TNBC) is a subtype of breast cancer that presents significant therapeutic challenges due to the absence of estrogen receptor (ER), progesterone receptor (PR), and human epidermal growth factor receptor 2 (HER2) expression. As a result, conventional hormonal and targeted therapies are largely ineffective, underscoring the urgent need for novel treatment strategies. γδT cells, known for their robust anti-tumor properties, show considerable potential in TNBC treatment as they can identify and eliminate tumor cells without reliance on MHC restrictions. These cells demonstrate extensive proliferation both *in vitro* and *in vivo*, and can directly target tumors through cytotoxic effects or indirectly by promoting other immune responses. Studies suggest that expansion and adoptive transfer strategies targeting Vδ2 and Vδ1 γδT cell subtypes have shown promise in preclinical TNBC models. This review compiles and discusses the existing literature on the primary subgroups of γδT cells, their roles in cancer therapy, their contributions to tumor cell cytotoxicity and immune modulation, and proposes potential strategies for future γδT cell-based immunotherapies in TNBC.

## 1 Introduction

Triple-negative breast cancer (TNBC) is a subtype of breast cancer distinguished by negative expression of estrogen receptor (ER), progesterone receptor (PR), and human epidermal growth factor receptor 2 (HER2) expression, comprising approximately 15–20% of all breast cancer cases (1). TNBC is notably aggressive, with a high rate of recurrence and metastasis, leading to poor prognoses and significant impacts on women’s physical and mental health (2–4). Its characteristics, including a high mutation rate, extensive T cell infiltration, and elevated expression of programmed death-ligand 1 (PD-L1), make it a focal point in immunotherapy research (5, 6). While immune checkpoint inhibitors (ICIs) have shown some effectiveness in treating TNBC (7), most patients with advanced disease do not respond well, complicating the search for new therapeutic targets (8). Currently, tumor-infiltrating lymphocytes (TILs)—including subsets of helper CD4+ cells, B cells, NK cells, γδT cells, and myeloid cells—are considered crucial biomarkers in tumor immunotherapy (9). Notably, γδT cell infiltration is regarded as the most favorable prognostic indicator (10). γδT cells originate from hematopoietic stem cells (HSCs) within the bone marrow and hold significant promise in the field of tumor immunotherapy. γδT cells are instrumental in tumor immunotherapy as they recognize and destroy tumor cells independently of specific antigen stimulation, unlike αβ T cells, which are part of adaptive immune responses (11). γδT cells serve as a crucial link between innate and adaptive immunity, functioning as the frontline defense against tumors and playing pivotal roles in tumor progression (12, 13). These cells possess innate-like receptors enabling rapid responses to diverse pathogens, facilitating early immune defense even in the absence of prior antigen exposure (14). Furthermore, γδT cells actively participate in tissue surveillance at barrier sites, contributing significantly to the maintenance of tissue homeostasis (15, 16). Depending on the tumor microenvironment, different γδT cell subsets can display either anti-tumor or pro-tumor activities. Predominantly, γδT cells eliminate tumor cells by recognizing tumor-associated antigens via their T cell receptors (TCR) and can also augment the anti-tumor efficacy of other immune cells by secreting cytokines or expressing co-stimulatory molecules (17). These characteristics also facilitate their integration into combination therapies, including chemotherapy, radiotherapy, or other immunotherapies, to enhance treatment outcomes.

Although γδT cells have demonstrated therapeutic potential in various cancers, their role and effectiveness in treating TNBC remain in the exploratory stage. This article reviews the current research on γδT cells in TNBC treatment, discusses their possible therapeutic mechanisms, and examines the integration of this unique immune cell type into existing treatment paradigms, offering new hope for TNBC patients. By extensively analyzing the biological characteristics of γδT cells, their molecular interactions with TNBC, and the latest developments in preclinical and clinical research, we can enhance our understanding of this strategy’s potential and challenges, thus paving the way for future research and the formulation of new treatment strategies.

## 2 Overview of γδT cells

### 2.1 Origin and distribution of γδT cells

T lymphocytes originate from pluripotent stem cells in the bone marrow. During embryonic and neonatal stages, some pluripotent or pre-T cells migrate to the thymus where, under the influence of thymic hormones, they differentiate and mature into immunologically active T cells. These mature T cells are then distributed to thymus-dependent areas of peripheral immune organs through the bloodstream and can recirculate through lymphatic vessels, peripheral blood, and tissue fluid, performing cellular immunity and immune regulation functions (18, 19). γδT cells, unique innate immune cells characterized by the expression of the γδ heterodimer T cell receptor, are relatively rare, constituting only 1% to 5% of peripheral blood T lymphocytes and are primarily found in mucosal tissues such as the skin, respiratory tract, digestive tract, and uterus (20). Human γδT cells originate in the medulla of the normal fetal thymus at 7–8 weeks, undergoing a developmental process similar to αβT cells, which includes functional TCR expression and negative selection to achieve self-tolerance. Unlike αβT cells, some γδT cells do not undergo positive selection, making them unrestricted by Major histocompatibility complex (MHC) in their antigen recognition and killing capabilities (21, 22). Various functional characteristics of γδT cells start to form in the thymus and gradually mature in the periphery. In the thymus, precursor cells differentiate into γδTCR+ thymocytes, which then exit to join the peripheral circulation as circulating γδT cells. These cells enter peripheral lymphoid organs and continue to develop under the influence of various hormones released by the thymus until they acquire the capabilities of mature immune cells (23, 24).

### 2.2 Genetic characteristics of γδT cells

T cells are classified into αβT cells and γδT cells based on the type of TCR expressed. γδT cells are a subpopulation of T cells characterized by their γ and δ chains in the T cell receptor, comprising 0.5–5% of all T cells. Unlike αβ T cells, which rely on the recognition of target antigens presented on MHC molecules by the αβ TCR to develop and function, γδT cells operate in an MHC-independent manner. αβ T cells differentiate into effector cells upon recognizing peptide-MHC (pMHC) complexes, enabling cytotoxic activity or cytokine production to defend against pathogens and tumors (22). In contrast, γδT cells do not require antigen processing and presentation by antigen-presenting cells (APCs) for activation, allowing for rapid early immune responses (25). γδT cell effector functions are activated by TCRs and natural killer receptors (NKRs) in response to stress-induced self-ligands (26). Moreover, similar to conventional αβ T cells, γδT cells can differentiate into various effector profiles and produce different chemokines and cytokines, including IFN-γ, TNF-α, IL-17, IL-21, and IL-22 (27). Additionally, human γδT cells may possess antigen-presenting capabilities; for instance, blood Vγ9Vδ2 T cells can respond to microbial and tumor signals and initiate CD4+ and CD8+ T cells, akin to dendritic cells (DCs) (28).

### 2.3 Classification and biological characteristics of γδT cells

γδT cells are primarily classified into three main subgroups based on the TCRδ chain: Vδ1T cells, Vδ2T cells, and Vδ3T cells (29, 30). Vδ1T and Vδ3T cells are predominantly found in mucosal and tissue environments, such as the skin, intestines, liver, and spleen. Vδ1T cells demonstrate significant anti-tumor effects in conditions such as colorectal cancer, multiple myeloma, and chronic lymphocytic leukemia (31, 32), yet they can also exert potent immunosuppressive effects when infiltrating tumors (33). The role of Vδ3T cells in tumors remains less understood (34). Vδ2T cells, the most abundant subgroup, represent 50%-90% of all γδT cells in peripheral blood and typically pair with Vγ9 TCR to form Vγ9Vδ2T cells, frequently utilized in clinical settings. γδT cells exhibit robust anti-tumor activities, making them valuable in adoptive immunotherapy for cancers. They are also capable of eliminating cancer stem cells in various tumors, including colon cancer, ovarian cancer, and neuroblastoma (35–38).

γδT cells are classified based on the expression of surface markers CD27 and CD45RA into four types: naive (CD27+CD45RA+, Tnaive), central memory (CD27+CD45RA−, TCM), effector memory (CD27−CD45RA−, TEM), and terminally differentiated (CD27−CD45RA+, TEMRA). Tnaive and TCM γδT cells, primarily located in peripheral lymphoid organs, lack immediate effector functions, whereas TEM and TEMRA γδT cells, commonly found at inflammation sites, exhibit immediate effector functions, including cytokine secretion and cytotoxic activities (39). Furthermore, γδT cells are also categorized based on function into γδT1, γδT17, follicular helper (γδTfh), regulatory (γδTreg), and memory γδT cells (40). Notably, γδT1 cells produce IFNγ and are recognized as positive prognostic markers in cancer (41). γδT17 cells secrete IL-17, which may promote tumor progression (42), while γδTreg cells, prevalent in TILs across various cancers, exert immunosuppressive effects (43). An understanding of the classification and biological characteristics of γδT cells is crucial for developing effective immunotherapies that leverage their potential.

## 3 Recognition pathways and functions of γδT cells

### 3.1 T Cell receptor-mediated recognition pathway

**Figure 1:**
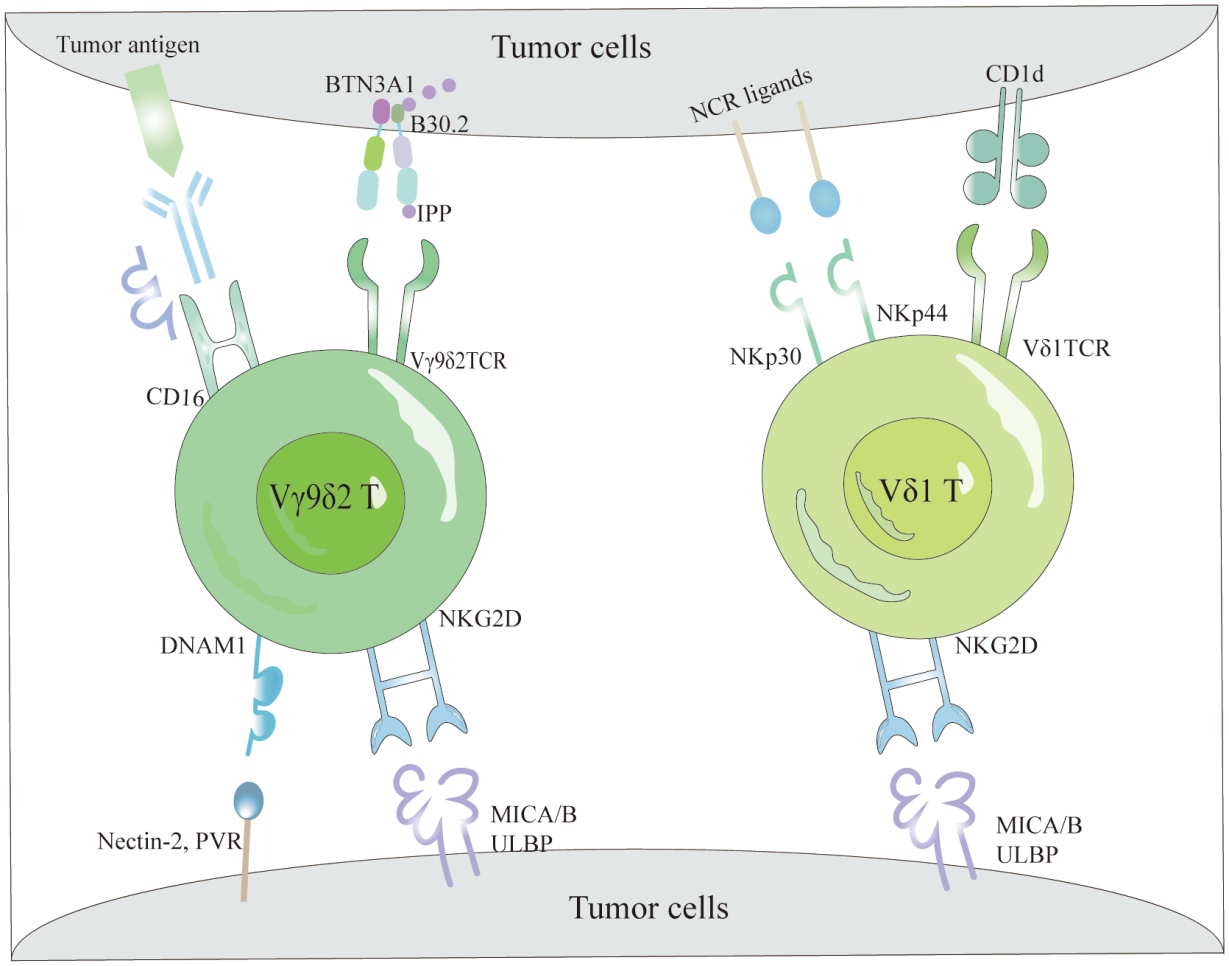
The tumor cell recognition of γδT cells. γδT cells recognize tumor cells based on the expression of γδTCRs and NKRs. Specifically, Vγ9Vδ2 TCR detects elevated intracellular levels of phosphorylated antigens, such as IPP, via BTN3A1, facilitating efficient tumor cell elimination. Additionally, they interact with tumor cells through the binding of NKG2D and DNAM-1 receptors to their respective ligands (MICA/B and ULBPs). Conversely, Vδ1 T cells recognize lipid antigens presented by CD1d via their Vδ1 TCR and utilize NKG2D and NCRs (NKp30 and NKp44) along with their corresponding ligands for tumor cell recognition. Moreover, Vγ9Vδ2 T cells effectively target and eradicate tumors via the CD16-mediated ADCC mechanism.

γδT cells identify various antigens through a TCR-dependent mechanism to detect and activate against tumor cells (**Figure 1**). Specifically, the Vγ9Vδ2TCR recognizes non-peptide phosphoantigens (P-Ag) such as microbial-derived (E)-4-hydroxy-3-methyl-but-2-enyl pyrophosphate (HMBPP), an intermediate in the non-mevalonate pathway of isoprenoid biosynthesis, which is a potent activator of γδT cells (44, 45). Similarly, host-derived isopentenyl pyrophosphate (IPP) acts as a P-Ag and stimulates Vγ9Vδ2T cell responses. Bisphosphonates inhibit the farnesyl pyrophosphate synthase (FPPS) in the isoprenoid biosynthesis pathway in target or APCs, causing IPP and its metabolites to accumulate and be targeted by Vγ9Vδ2T cells (46, 47). Mookerjee-Basu et al. and Scotet et al. have documented that tumors sensitive to Vγ9Vδ2 display and interact with a complex similar to mitochondrial ATP synthase, specifically F1-ATPase (48, 49). The activation of Vγ9Vδ2T cells is further enhanced in the presence of apolipoprotein (apoA-I), as F1-ATPase can complex with apoA-I to present phosphoantigens recognizable by Vγ9Vδ2 TCR (50). Research indicates that detecting P-Ag in target cells necessitates a surface protein with intracellular and extracellular domains, specifically butyrophilin 3A1 (BTN3A1 or CD277), which binds HMBPP to its intracellular B30.2 domain. This binding induces an extracellular conformational change, facilitating the recognition of target cells by Vγ9Vδ2T cells through inside-out signaling (51, 52). Additionally, few ligands for Vδ1 TCR have been identified, with lipid antigens presented by the MHC-like molecule CD1d binding to Vδ1 TCR (**Figure 1**), a relationship elucidated by the crystal structure of the Vδ1 TCR with CD1d-sulfatide (53). This capability of γδT cells to recognize tumor cells via TCR underscores their integral role in both innate and adaptive immunity.

### 3.2 NK cell receptor pathway

The mechanisms by which γδT cells identify tumor cells are not limited to TCR interactions but also include their reliance on NKRs (54) (**Figure 1**). These cells primarily identify tumor cells through NKRs such as NKG2D, DNAM-1, NKp30, NKp44, and NKp46, which bind to specific ligands present on tumor cells (55). NKRs not only regulate the activation and function of NK cells but also facilitate immune surveillance by γδT cells, enabling the distinction between transformed and infected cells. NKG2D, a C-type lectin receptor, binds ligands that are typically absent in most normal tissues but are overexpressed on tumor cells, thereby enabling γδT cells to recognize and eliminate tumor cells (56). Identified ligands for NKG2D in human cells include MHC class I chain-related proteins (MICA/MICB) and six UL16-binding proteins (ULBP1–6) (57). Contrary to previous beliefs that natural cytotoxicity-triggering receptor (NCRs) were exclusive to NK cells, recent data reveal their presence in T cells and NK-like cells (58, 59). Although Vδ1+ and Vδ2+ cells naturally lack NCR expression, it can be selectively enhanced in Vδ1+ cells through AKT-dependent signaling triggered by γc cytokines (IL-2 or IL-15) and TCR stimulation (60). The NCRs expressed in Vδ1+ γδT cells (**Figure 1**), predominantly NKp44 and NKp30, endow these cells with heightened abilities for targeted cytotoxicity against tumor cells and for secreting IFN-γ (55, 61).

### 3.3 CD16 pathway

One pathway through which Vγ9Vδ2T cells exert their anti-tumor effects involves CD16, also known as FcγRIII (**Figure 1**), a low-affinity type III receptor that specifically binds to the Fc portion of Immunoglobulin G (IgG) (62). This receptor mediates antibody-dependent cellular cytotoxicity (ADCC) and cytokine production, including TNF-α (63). Research indicates that Vγ9Vδ2T cells treated with zoledronic acid and IL-2 can express CD16 (64). The interaction of CD16 with the Fc portion enables Vγ9Vδ2T cells to detect and destroy tumor cells expressing IgG through ADCC activation (65, 66). Depending on the presence or absence of CD16 expression, Vγ9Vδ2T cells are classified into two types: CD16− and CD16+. The CD16− subset produces higher cytokine levels, expresses fewer killer inhibitory receptors (KIRs), and exhibits lower cytotoxicity, while the CD16+ subset has higher KIR levels and significant direct cytotoxic capabilities (67). The presence of CD16 enhances the recognition capabilities of Vγ9Vδ2T cells against IgG-expressing tumor cells, particularly in the CD16+ subgroup, which shows enhanced direct cell-killing ability.

## 4 Anti-tumor effects of γδT cells

**Figure 2:**
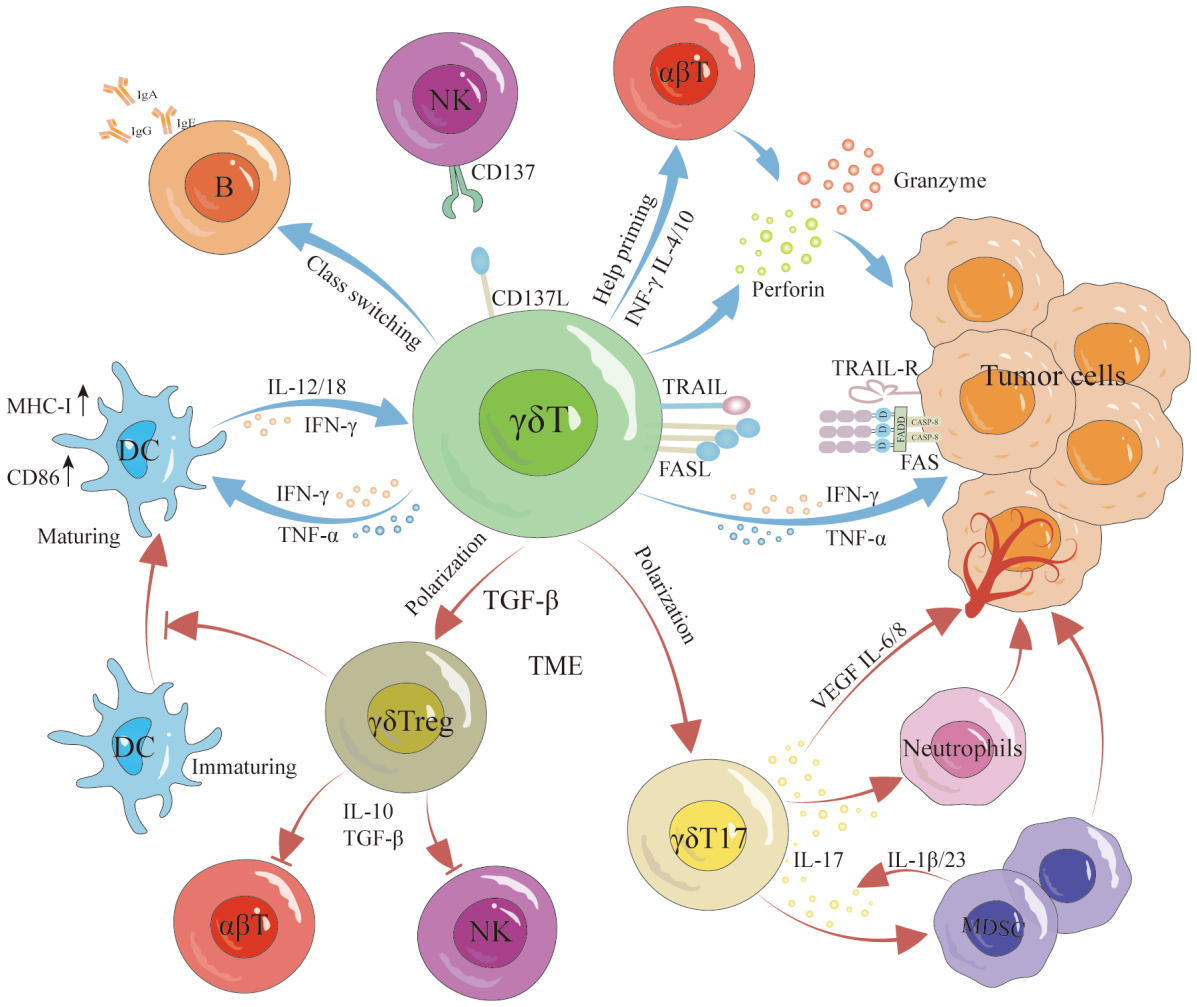
Antitumor and protumor functions of γδT cells. γδT cells exert antitumor effects through various pathways, involving both direct and indirect mechanisms. Direct antitumor effects include lysing the tumor via the perforin-granzyme pathway, Fas/FasL pathway, and TRAIL signaling. Furthermore, they indirectly hinder tumor growth by promoting DC maturation, inducing B cell activation, enhancing αβT cell activation and proliferation, and augmenting NK cell-mediated cytotoxicity against malignant cells. However, it is noteworthy that γδT cells can differentiate into γδTreg cells and γδT17 cells, promoting tumor growth. Specifically, γδTreg cells secrete IL-10 and TGF-β to suppress αβT cell and NK cell responses to tumors while concurrently inhibiting DC maturation. γδT17 cells promote tumorigenesis by secreting IL-17 to recruit MDSCs and stimulate the production of VEGF, IL-6, and IL-8, inducing angiogenesis and expediting tumor growth.

γδT cells have the unique capability to recognize and destroy tumor cells without relying on traditional antigen presentation mechanisms, which is particularly advantageous in targeting tumors like TNBC that lack specific antigen presentation. γδT cells can directly lyse tumor cells via two independent pathways (**Figure 2**): firstly, by secreting perforin and granzymes (68, 69); secondly, by inducing cell death through the Fas/FasL pathway and tumor necrosis factor-related apoptosis-inducing ligand (TRAIL) (70, 71). On their surface, γδT cells express FasL, which can trigger programmed apoptosis in tumor cells by forming a Fas trimer upon binding to Fas (72). This interaction leads to the activation of the death effector domain (DED) and Fas-associated death domain-containing protein (FADD), subsequently activating downstream caspases that result in cellular destruction and death (73–75). Similarly, TRAIL induces apoptosis via caspase activation through FADD (71, 75). γδT cells also indirectly exert anti-tumor effects by activating other immune cells and possess antigen-presenting capabilities that stimulate the activation and proliferation of αβT cells (76, 77) (**Figure 2**). They promote the proliferation and differentiation of CD8+ T cells and regulate TNF-α and IFN-γ secretion, enhancing tumor clearance rates (78, 79). Additionally, research by Bansal et al. found that γδT cells expressing CXCL13 and CXCR5 demonstrate follicular helper T cell (Tfh) characteristics, particularly in the presence of IL-21, which aids B cell support (80, 81). Caccamo et al. discovered that Vδ2γδT cells can activate B cells (**Figure 2**), leading them to produce significant amounts of immunoglobulins even without antigen stimulation, and facilitate the formation of germinal centers (82, 83). Moreover, Maniar et al. showed that zoledronic acid-activated γδT cells enhance NK cell-mediated cytotoxicity (**Figure 2**) against tumor cells, a process reliant on the interaction between CD137 ligand on γδT cells and CD137 on NK cells (84). Furthermore, γδT cells can modulate the production of cytokines such as interferon-γ by NK cells, influenced by DC-like cells (85).

The interaction between γδT cells and DCs is reciprocal (**Figure 2**). γδT cells facilitate the maturation of DCs, while mature DCs trigger the activation and proliferation of γδT cells, thereby enhancing their anti-tumor capabilities (86). Typically, Fas/FasL mediates cell apoptosis; however, notably, DCs express high levels of the Fas inhibitor cFLIP. Elevated cFLIP levels convert the pro-apoptotic signal of Fas into one that promotes dendritic cell maturation and function (87). Additionally, activated γδT cells secrete IFN-γ and TNFα, stimulating the expression of CD86 and MHC-I molecules on DCs surfaces, which supports further DCs maturation (88). Conversely, mature DCs boost γδT cell proliferation and augment their cytotoxic and immunoregulatory abilities by secreting cytokines such as IL-1β, IL-12, IL-18, IFN-γ, and TNF-α (89). DCs also facilitate contact-dependent activation of γδT cells through CD86-CD28 interactions and the expression of specific γδT cell activation ligands, like P-Ag (90). These intricate interactions underscore the versatile role of γδT cells in modulating anti-tumor immune responses.

## 5 The protumor function of γδT cells

While γδT cells serve an effector role in anti-tumor immune responses, their function is frequently inhibited by the tumor microenvironment (TME), which may even lead these cells to differentiate into subgroups that foster tumor progression. Research has demonstrated that tumor-infiltrating γδT17 cells (**Figure 2**) exacerbate tumor development by secreting IL-17 (91). IL-17, a pro-inflammatory cytokine, promotes chronic tissue inflammation, thus aiding tumor progression. In cancers such as pancreatic, liver, non-small cell lung, and breast cancer, elevated IL-17 levels correlate with increased metastasis and poor prognosis (92). IL-17 enhances tumor progression through several mechanisms: it activates the PI3K/AKT signaling pathways to stimulate tumor cell proliferation (93), induces the production of matrix metalloproteinases (MMPs) which degrade the extracellular matrix, facilitating tumor cell invasion and distant metastasis (94), and promotes angiogenesis by stimulating angiogenic factors including VEGF, IL-6, and IL-8 (95, 96). Furthermore, IL-17 recruits myeloid-derived suppressor cells (MDSCs) into the tumor, sustaining an immunosuppressive TME (97, 98) (**Figure 2**). A study by Ma and colleagues revealed that MDSCs could also drive γδT cells to produce IL-17 via IL-1β and IL-23, perpetuating a malignant cycle of tumor progression (99).

γδT cells potentially regulate tumor immune responses. In breast cancer, Vδ1+ γδT cells infiltrate and inhibit the proliferation of inactive T cells and the functionality of effector CD4+ and CD8+ T cells. They also suppress the maturation of DCs (**Figure 2**) and the proliferation of anti-tumor Vδ2+ cells, fostering an immunosuppressive environment (100). Furthermore, studies have shown that γδT cells can differentiate into FoxP3+ γδTreg cells under the influence of γδTCR monoclonal antibodies and TGF-β (101). These γδTreg (**Figure 2**) cells, similar to traditional Treg cells, secrete inhibitory cytokines such as IL-10 and TGF-β, which suppress the immune response of αβT cells and NK cells against tumors. Specifically, breast cancer-derived γδTreg cells can induce immunosenescence in naive T cells, effector T cells, and DCs. They also inhibit the proliferation of T cells from human peripheral blood mononuclear cells (PBMCs) (102), and by inducing CD86/CTLA-4 and PD-L1/PD-1 interactions, they alter the tumor environment’s structure and reduce effector T cell activity (103). It is clear that the TME influences the phenotype and function of γδT cells, with γδT17 cells exacerbating inflammation, promoting angiogenesis, and recruiting MDSCs and other inhibitory cells, thereby enhancing tumor progression. Correcting the TME and reversing the negative regulatory effects of tumor-infiltrating γδT cells are critical for leveraging γδT cells in cancer immunotherapy.

## 6 The role of γδT cells in TNBC

γδT cells, as crucial components of TILs, play a significant role in regulating tumor immune responses. Notably in TNBC, research by Chabab et al. has revealed that γδT cell infiltration often exceeds that in other breast cancer (BC) types, a phenomenon closely linked to TNBC’s higher mutation rate (104). Multiple studies suggest that the presence of TILs in TNBC often correlates with a more favorable prognosis (105, 106). Wu et al. have shown that the abundance of Vδ1+ γδT cells, rather than the total number of γδT cells, is critical in determining the treatment response in TNBC patients. These infiltrating Vδ1+ T cells, with their cytotoxic capabilities and ability to produce IFN-γ, operate through an intrinsic mechanism as they respond to MICA and cytokines IL-12 and IL-18. Further research indicates that the density of Vδ1+ T cells positively correlates with patients’ progression-free survival (PFS) and overall survival (OS) (107). Craven et al.’s data analysis also supports this finding, linking γδTILs with prolonged OS (108). Conversely, Janssen et al. highlight that in TNBC, Vδ2+ T cells are the predominant γδTILs subgroup, actively contributing to anti-tumor effects by secreting IFN-γ and TNF-α. Their studies also reveal that γδTILs are not sources of IL-17 in TNBC, unlike in colorectal cancer, suggesting that TNBC’s γδTILs may not depend on IL-17 for promoting tumor growth (109). Moreover, evidence suggests that γδT cells may facilitate breast tumor development through their immunoregulatory functions, correlating with poor prognosis in breast cancer. Within the TME of human breast cancer, a minority (<20%) of Vδ1 T cells that express CD73 and can produce IL-10, adenosine, and IL-8 exhibit immunosuppressive effects (110). These findings suggest that while some γδT cell subgroups are immunosuppressive, their impact is often masked by those with anti-tumor activity. Consequently, further investigation into the role of γδT cells in TNBC or its subtypes, and their influence on disease progression and treatment responses, is critically important.

## 7 The therapeutic potential of γδT cells in TNBC

TNBC presents significant diagnostic and treatment challenges due to its ambiguous biological characteristics. The IMpassion130 study marked a pivotal shift into the era of immunotherapy for breast cancer, making TNBC the most extensively studied cancer type in this domain (111). Recently, enhanced insights into the tumor microenvironment and immune evasion mechanisms have established immunotherapy as a viable approach for TNBC. γδT cells, which recognize tumor antigens without MHC restrictions and exhibit potent cytotoxic effects, can be substantially expanded *in vitro* and *in vivo*, demonstrating significant potential in tumor immunotherapy. The presence of TILs in TNBC has been linked to favorable prognoses, highlighting that adoptive cell therapy (ACT) provides new therapeutic avenues. ACT primarily encompasses therapies such as chimeric antigen receptor (CAR)-T, TCR, and TILs therapies, which all operate on similar principles (112).

The induction and adoptive transfer of γδT cells, particularly targeting Vδ2 and Vδ1 subtypes, represent a promising avenue in cancer immunotherapy research. Commonly, the combination of P-Ag such as BrHPP and HMBPP or nitrogen-containing bisphosphonates (N-BP) like zoledronic acid (ZOL) with IL-2 is utilized for both *in vivo* and *in vitro* expansion of Vδ2 γδT cells. This approach has been extensively implemented and clinically validated for therapeutic safety (21, 113–115). ZOL not only facilitates the transformation of Vδ2T cells into TEM phenotypes but also significantly boosts their cytotoxic capabilities, thus enhancing tumor suppression (116). *In vivo*, Vγ9Vδ2 T cells stimulated by P-Ag or N-BP also demonstrate the capacity to target and eliminate multiple tumor cells. However, the clinical response rate is typically lower compared to that observed in cases of overt metastasis (117, 118).In the context of neoadjuvant therapy for breast cancer, combining letrozole with ZOL to expand γδT cells *in vivo* has demonstrated substantial patient benefits (119). Additionally, ex vivo expansion of γδT cells for adoptive immunotherapy has shown notable anti-tumor effects in animal models (120). Vitamin C (VC) and its derivatives also positively influence the proliferation and activation of Vδ2T cells, particularly at high doses, where VC augments the cytotoxic effects of CD8+ T cells and synergistically boosts the efficacy of immunotherapy alongside immune checkpoint inhibitors (121–123). A novel protocol for Vδ2 T cell expansion developed by Xu et al., integrating ZOL, IL-2, IL-15, and VC, has proven effective in enhancing cell proliferation, differentiation, and cytotoxicity, significantly curtailing tumor growth and extending survival in mice (124).

Recent advancements in cancer immunotherapy have introduced an approach involving Delta One T (DOT) cells derived from Vδ1+ T cells. These cells are activated through TCR agonists and cytokines, resulting in substantial proliferation (12, 125). DOT cells have shown promising therapeutic effects against various tumor types, an outcome further enhanced by increased expression of NKp30, NKp44, NKG2D, and DNAM1 (126, 127). Additionally, Raute et al. have found that primarily expanded Vδ1+ and Vδ2+ T cells can target triple-negative breast cancer stem cells (BCSC) derived from patients. However, these BCSCs may differentiate *in vivo* into cells that lack stem cell-like properties and γδT cell activation ligands, thereby escaping effective γδT cell-mediated destruction. Nonetheless, γδT cells can still marginally kill these differentiated cells *in vivo* by recognizing P-Ag. Significantly, the cytotoxic effect is enhanced by ZOL, suggesting that a combination of γδT cells and ZOL might represent an effective strategy against both triple-negative BCSCs and non-stem tumor cells (128). In conclusion, the expansion of γδT cells offers new hope for treating patients with TNBC. Although this therapy remains under research, its potential for broad application in TNBC treatment holds significant promise as an effective therapeutic option.

CAR-T cell therapy, a form of adoptive cell therapy, merges the antigen specificity of antibodies with the effector functions of T cells, showing considerable potential to improve survival rates in TNBC patients (129). Upon reintroduction into the patient, CAR-T cells initiate cytotoxic immune responses by recognizing tumor-associated antigens. Initially employed in refractory hematologic malignancies (130), this technology has been extensively studied in TNBC, targeting antigens like ROR1, c-Met, EGFR, FRα, and MUC1. These targets provide specific foci for CAR-T cell therapy. However, the efficacy in solid tumors is often hampered by challenges such as tumor antigen heterogeneity, the immunosuppressive tumor microenvironment, and the limited infiltration and persistence of CAR-T cells within tumors (131, 132). Moreover, research has also ventured into expressing CARs on other effector cells like CAR-γδT cells, which specifically recognize and target tumor cell surface antigens, delivering a cytotoxic response (133). Capsomidis et al. have shown that CAR-γδT cells not only migrate efficiently to tumor sites but also exhibit strong cytotoxicity directed by specific tumor antigens (134). Demonstrated in xenograft mouse model studies, these cells exhibit significant anti-tumor activity both *in vitro* and *in vivo*, suggesting they could form a novel treatment approach for TNBC (135).

Abnormal signaling of immune checkpoint molecules has been observed to disrupt the normal function of the TCR and alter the phosphorylation levels of intracellular proteins via ITIM motifs and SHP-1/2. Consequently, this interference inhibits the proliferation and activation of γδT cells, leading to a reduction in their cytotoxicity (136). Therefore, the combined application of γδT cells and ICIs may emerge as an effective strategy to enhance the therapeutic efficacy of TNBC. Additionally, bispecific antibodies have demonstrated potential in augmenting the efficacy of γδT cell immunotherapy by significantly enhancing cytotoxicity through the fusion of tumor-binding and T-cell splicing structural domains (137). Oberg et al. reported that the administration of γδT cells expanded *in vitro* with specific bispecific antibodies effectively slowed the growth of pancreatic and colon cancers in preclinical models (138, 139). Additionally, bispecific molecules (GABs) linking the extracellular domain of the tumor-reactive Vγ9Vδ2 TCR to a CD3 binding structure have been shown to promote T-cell infiltration into the tumor microenvironment, thereby inhibiting tumor growth *in vivo (* 140). Thus, the bispecific splicer holds great potential as a form of γδT cell-based immunotherapy. If successful in clinical trials, this treatment could offer a powerful and relatively inexpensive therapeutic option for TNBC patients. Another innovative approach involves transducing αβ T cells with a high-affinity Vγ9Vδ2 TCR, termed T cells with defined γδTCR (TEG) (12). TEG demonstrates the ability to target a wide array of solid and hematological tumors and, in addition to exerting cytotoxic effects, exhibits paracrine activity that induces functional maturation of dendritic cells (14). Consequently, TEG can effectively target a broad spectrum of tumor cells owing to the wide reactivity of the Vγ9Vδ2 TCR, thereby addressing the limitations of low persistence and impaired activation of γδT cells within the tumor microenvironment (12, 125). These novel therapeutic strategies instill hope for patients with triple- TNBC by expanding treatment options and possibilities for the future.

## 8 Major challenges and clinical implications of utilizing γδT cells

One of the major challenges for γδT cells is their scarcity in the immune system, as they represent only a small fraction of the total T cell population (20). This limited presence hampers the ability to fully exploit their therapeutic potential, especially when compared to the more abundant αβ T cells. Additionally, the specific tissue distribution of γδT cells, predominantly located in the peripheral regions of non-lymphoid tissues, complicates their accessibility and clinical application. Despite considerable efforts, expanding various clones of γδT cells to clinically relevant numbers remains a significant obstacle to their widespread use in cellular immunotherapy (141). However, the Vδ1 subpopulation has been shown to predict a favorable prognosis in triple-negative breast cancer, as supported by protein or gene level analyses (9, 107). Another significant challenge is the limited role of γδT cells in the TME. Although several studies have shown that γδT cells can modulate immunosuppressive cells within the TME, the scarcity of nutrients, the presence of suppressor molecules, and hypoxia may still constrain their therapeutic potential (142, 143). Suppressive molecules produced by tumor cells and other cells in the TME, such as TGF-β (144), prostaglandin E2 (PGE2) (145), adenosine (146), and soluble NKG2D ligands (147), can interfere with γδT cell proliferation and function.

Despite these challenges, ongoing clinical trials are assessing the safety and antitumor efficacy of γδT cells. However, the clinical utility of Vγ9Vδ2 T cells may be hindered by susceptibility to T cell exhaustion and activation-induced cell death (AICD) (148). Moreover, in rare cases, stimulation of γδT cells with phosphoantigens and ZOL may lead to adverse effects including fever, fatigue, eosinophilia, thrombosis, elevated liver transaminases, hyperglycemia, gastritis, musculoskeletal pain, and nephrotoxicity (149, 150). Thus, the safety and tolerability of γδT-cell therapies require meticulous consideration and monitoring throughout design and implementation. In conclusion, despite numerous challenges, γδT cell therapy holds significant promise as a novel immunotherapeutic approach. By comprehensively investigating the tumor microenvironment, devising effective therapeutic strategies, and employing advanced immunological techniques to expand and activate γδT cells, the efficacy of this therapy can be enhanced to yield improved clinical outcomes for patients with tumors.

## 9 Summary and future perspectives

TNBC, characterized by the absence of ER, PR, and HER2 expression, limits patients’ options for hormone or targeted therapies, thereby necessitating new treatment strategies. γδT cells, key components of immune defense, can target and destroy tumor cells independently of traditional MHC-mediated antigen presentation, thereby exerting significant anti-tumor effects. However, their potential pro-tumor activities, including suppressing anti-tumor responses, enhancing tumor angiogenesis, and secreting IL-17, are subjects of ongoing debate (68, 151). Although the tumor microenvironment is thought to recruit numerous γδT cells that may promote tumor progression, single-cell RNA sequencing from fresh breast cancer tissues and patients’ peripheral blood reveals that γδT cells generally correlate with favorable clinical outcomes, with tissue-infiltrating γδT cells being more active and cytotoxic than their blood counterparts (152). Recent mouse studies have noted pro-tumor and pro-metastatic effects in γδT cells producing IL-17, although such cells are rare in humans. Evidence suggests that γδTILs contribute minimally to IL-17 secretion compared to Th17 and CD4+ T cells in the TME (109). This underscores the potential of γδT cell-based immunotherapy as a novel strategy for breast cancer treatment. Research indicates that γδT cells can inhibit TNBC progression through direct cytotoxic actions and by modulating immune responses. The presence of γδTILs in the TNBC microenvironment is strongly associated with favorable patient prognoses, underscoring their vital role in anti-tumor immunity. While various methods to expand γδT cells have shown promise in anti-tumor therapy, specific studies on their application in TNBC remain limited. Furthermore, enhancing the activity or specificity of γδT cells through CAR technology presents opportunities to improve their therapeutic potential in TNBC treatment.

Future research will persist in investigating the use of γδT cells in treating TNBC, with a particular emphasis on effectively expanding and activating these cells, overcoming the immunosuppressive tumor microenvironment, and enhancing their tumor-homing capabilities. Moreover, the use of engineered γδT cells, such as CAR-γδT cells and TEGs, either alone or in combination with checkpoint inhibitors, holds promise for enhancing the response rate and anti-tumor effects of γδT cell therapy. Considering the complexity and therapeutic challenges of triple-negative breast cancer (TNBC), researchers must explore novel methods or techniques to achieve effective expansion of both Vγ9Vδ2 T cells and Vδ1 T cells. Specifically, the advancement of CAR-γδT cell therapy necessitates additional clinical data to support its use in TNBC treatment. As our understanding of the tumor microenvironment and immune evasion mechanisms expands, coupled with cutting-edge immunotherapies, TNBC treatment is poised to become increasingly personalized, significantly enhancing patient prognosis and quality of life.

## Author contributions

WL: Conceptualization, Visualization, Writing – original draft, Writing – review & editing. XZ: Writing – original draft, Writing – review & editing. CR: Writing – original draft, Writing – review & editing. SG: Writing – review & editing. QH: Writing – original draft, Writing – review & editing. ML: Writing – original draft, Writing – review & editing. XL: Funding acquisition, Project administration, Resources, Supervision, Writing – review & editing.
